# Saguaro (*Carnegiea gigantea*) Mortality and Population Regeneration in the Cactus Forest of Saguaro National Park: Seventy-Five Years and Counting

**DOI:** 10.1371/journal.pone.0160899

**Published:** 2016-08-09

**Authors:** Thomas V. Orum, Nancy Ferguson, Jeanne D. Mihail

**Affiliations:** 1 Sweetwater Center, Tucson, Arizona, United States of America; 2 Division of Plant Sciences, University of Missouri, Columbia, Missouri, United States of America; University of Arkansas, UNITED STATES

## Abstract

Annual census data spanning seventy-five years document mortality and regeneration in a population of saguaro cactus (*Carnegiea gigantea*) in the Cactus Forest of the Rincon Mountain District of Saguaro National Park near Tucson, AZ. On 6 four-hectare plots, each saguaro was censused and a methodical search for new saguaros was conducted annually each year from 1942 through 2016, with the exception of 1955. Regeneration has been episodic with 828 plants established from 1959 through 1993 compared with 34 plants established between 1942 and 1958 and only three plants established after 1993. The years preceding 1959 and following 1993, include some of the driest decades in centuries in southern Arizona. While woodcutting and cattle grazing are believed to be among the causes of decades of failed regeneration prior to 1958, neither of these factors contributed to the failed regeneration following 1993. The height structure of the population from 1942 to 2016 shifted dramatically from a population dominated by large saguaros (> 5.4 m tall) in the first three decades of the study to a population dominated by small saguaros (< 1.8 m tall) in the most recent two decades. Mortality is shown to be strongly age dependent. In the year following the 2011 catastrophic freeze, 21 of 59 plants older than 80 years died compared with zero deaths in 270 plants between the ages of 29 and 80 years. Saguaros under 40 years old, growing under small shrubs or in the open, have a lower probability of survival than better protected saguaros. Long-term population monitoring is essential to understanding the complex impacts of human and environmental factors on the population dynamics of long-lived species.

## Introduction

Beginning with Forrest Shreve’s classic studies of the giant saguaro cactus (*Carnegiea gigantea* [Engelm.] Britton & Rose) at the Carnegie Institution Desert Laboratory on Tumamoc Hill in Tucson, Arizona [1,2], we have come to understand that saguaro population regeneration is often episodic [3–6]. Previously published studies have been based on single or infrequent observations of the height structure of the population from which saguaro ages and establishment years are estimated [4,6,7]. Episodes of regeneration are then reconstructed by comparing age distributions with survivorship curves. The present study is unique because it is based on annual observations that completely encompass one episode of population regeneration. This episode is examined in detail using data from 75 years of consecutive annual censuses of a saguaro population in the Cactus Forest of Saguaro National Park, Rincon Mountain District, Tucson, Arizona.

The Cactus Forest at Saguaro National Monument was in its prime in 1940, just seven years after the monument’s founding, when caretakers began to notice high mortality of saguaros. Concern for the saguaro population led to the establishment in 1941 of a square mile (256 ha, 640 acres) section of the Cactus Forest devoted to researching saguaro mortality [8]. In addition to concern about mortality, researchers were also concerned about population regeneration [9]. Mielke [10] observed that there were almost no young saguaros in the study area and described the habitat in the Cactus Forest as practically bare of vegetation except for the large saguaros. This was attributed to overgrazing, woodcutting and high rodent populations. The plots containing the population reported on here were selected in 1945 from the initial square mile section for ongoing study. The 1950s were a time of severe, extended drought–later characterized as the worst drought in the southwestern United States in 400 years [11]. The lack of regeneration led researchers using these plots in the 1950s and early 1960s to study the biology of saguaro population regeneration including pollination [12,13], seed germination [14] and the influence of shade and other factors on seedling survival [15].

Saguaros begin to produce fruit at 30 to 45 years of age and have a lifespan estimated to be between 125 to 175 years [7]. As a result, some saguaros are capable of producing seed for over a century. A long reproductive period is one mechanism by which a species can persist in an environment such as the Sonoran Desert, where conditions conducive to seedling establishment can vary significantly. The vegetation community in the Sonoran Desert depends on both winter rains and summer monsoon thunderstorms [16]. The amount of rain produced during these seasons can vary at the scale of decades [11]. Saguaro populations survive these precipitation patterns in the Sonoran Desert because of their long reproductive period and prodigious seed production capability.

Because the saguaro is an icon of both the Sonoran Desert and the state of Arizona, there is considerable popular interest in the health of the saguaro population in the face of changing climatic conditions. The current report represents a detailed study of saguaro mortality and regeneration for a period of seventy-five years that includes one episode of regeneration spanning approximately three decades in one part of the Cactus Forest of the Rincon Mountain District of Saguaro National Park.

## Methods

### Ethics Statement

This study was carried out in the Rincon Mountain District of Saguaro National Park in Tucson, Arizona with the permission of the National Park Service. No samples were collected. The study was done in accordance with the conditions set forth in our research permit from the National Park Service. The current permit number is SAGU-2014-SCI-0001.

### History

**Fig 1:**
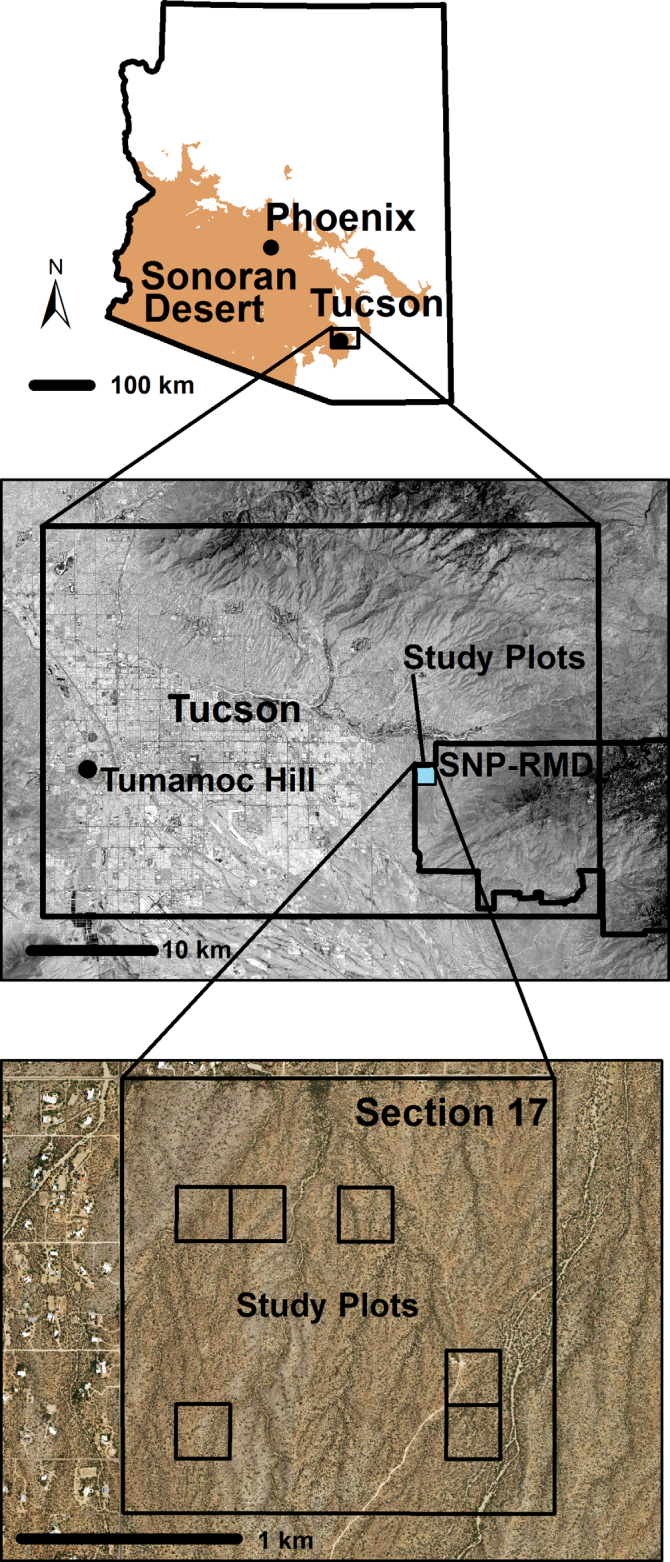
Location of study plots in the Rincon Mountain District of the Saguaro National Park (SNP-RMD) east of Tucson, AZ. Census plots are located in Section 17, Township 14S, Range 16E, Gila and Salt River Base and Meridian. The base map for the middle panel is a June 1, 2015 Landsat 8 image and the base map for the bottom panel is May 30, 2015 NAIP JPG2000 aerial image, both from the United States Geological Survey EROS data center. The illustration of the Sonoran Desert in the upper panel is derived from the Natural Vegetation layer available in the public domain section of the AZGEO Clearinghouse.

Between October 1940 and May 1941, University of Arizona engineering students surveyed the corners of sixty-four 4-hectare plots forming a grid in Section 17, Township 14 South, Range 16 East, at Saguaro National Monument (now Saguaro National Park, Rincon Mountain District) east of Tucson, Arizona (Fig 1). A numbered wooden stake was placed next to each of the 12,898 living saguaros in the 64 plots. Staking was completed in 1942. The thirty-two plots in the south half of the section were “treated” by removing individuals with black cortical rot and the thirty-two plots in the north half of the section were the untreated control. The plant removal treatment occurred fully only once, in 1941–42, and to a limited extent in 1943 [17]. World War II intervened and no treatments were carried out after 1943, but all plants in the 64 plots were observed annually. In 1945, the removal experiment was terminated. Six plots of the original sixty-four plots were selected for ongoing monitoring–three in the treatment area (south half) and three in the untreated area (north half) [17]. Each of the three treatment plots was paired with a control plot of similar saguaro population density in 1946. The saguaros in these six plots have been censused annually from 1942 to the present (except for 1955).

In any study spanning over seven decades, events will occur adding to the individuality of plots and this study is no exception. As noted above, three plots were “treated” in 1942, whereas three plots were not. The north half of section 17 (untreated) was privately owned until the 1970s, when it was purchased by the Monument. Cattle grazing occurred on all plots until the mid-1960s when a boundary fence excluded grazing from the southern plots. Grazing was excluded from the northern plots later. Saguaros were transplanted into one plot in 1958 [9]. The surviving transplants (approximately 48 plants in 19 clumps) are not included in this study because the focus is on a natural episode of establishment. Only recently have a few (10 of 48 in 2009) of the transplants begun to flower, so the transplants did not contribute seed to the episode of establishment reported here. A small picnic area in one of the southern plots has been expanded over the decades. Foot and horse trails pass through two of the plots. Here, we examine saguaro establishment and mortality occurring in the combined population of all six plots regardless of their individual histories.

### Habitat description

The saguaro population discussed here is located in the flats of the Rincon Mountain District of the Saguaro National Park. Steenbergh and Lowe [8,18] emphasize important differences in saguaro population structure and dynamics between rocky and non-rocky (flats) habitats in the Rincon Mountain and Tucson Mountain Districts of Saguaro National Park. Based on the age structure of the populations in the mid-1970s, they suggest that saguaro populations in the rocky habitats are more stable than populations in the flats. Population dynamics differ with climatic differences across the range of the saguaro and also with microhabitat differences relating to slope, aspect, soil type, and local features affecting water runoff and accumulation [6].

The flats where the plots are located consist of quaternary alluvial soil of the lower bajada of the Rincon Mountains. The landscape slopes downward gradually from south to north and is interspersed with a network of northwardly-flowing washes that are usually 2–4 m below grade and of varying widths (Fig 1). The resulting landscape consists of washes and ridges with dramatically different vegetation types. The ridges contain saguaro, foothill paloverde (*Parkinsonia microphylla* Torr.) and some mesquite (*Prosopis velutina* Woot.) whereas wash bottoms are dominated by mesquite, creosote bush (*Larrea tridentata* (DC) Coville), and ribbons of catclaw acacia (*Acacia* (*Senegalia*) *greggii* A. Gray) along watercourses, but contain few saguaro and paloverde.

### Annual census

The annual saguaro census consists of visiting each saguaro that was living in the previous year, assessing its condition, and noting mortality. In 1942, all plants were classified into five height classes: class I (0.0–1.8 m); class II (1.8–3.7 m); class III (3.7–5.5 m); class IV (5.5–7.3 m) and class V (>7.3 m). Plants were classified by height class again in 1960, and in many subsequent years. The height classes of plants were evaluated in several ways over the course of the study. In most years, when a plant appeared to be near a change in height class, we evaluated the height class by sighting from a distance using a 1.8 m reference standard placed next to the saguaro. A clinometer was used in 1983 and 1987 to measure all plants and check height classes. All plants were measured in 2011 with a fiberglass extension pole. Since 1968, we have counted the number of branches greater than approximately 10 cm diameter on each plant. Individual plants are identified based on their map location, height or height class, and individual marks (such as scars, bird holes, insect holes, rodent damage, etc.).

Each spring, typically in March through 1999 and in January through March thereafter, we methodically search for new plants. When found, we map them, measure them, describe any marks or scars, and note the nurse plant and the direction of the saguaro from the base of the nurse plant. Care is taken with descriptions and measurements of individuals to insure that newly discovered plants are found in subsequent years. In the 1990s the original maps were digitized and entered into an ArcInfo geographic information system (GIS), which has been maintained using Arc View 3.1 (Environmental Systems Research Institute [ESRI], Redlands, CA). After 1979, except for 1982, the height of every plant less than 1.8 m tall was measured to the nearest 0.5 cm every year.

### Estimating germination year

We estimated the year of germination for new plants found in our plots based on an estimate of the age of the plant in the year it was found. The standard way to estimate age of saguaros is to use a table relating height to age developed from growth measurements taken of plants of various heights [1,7,8,19]. We grouped cover plants for saguaros into three classes based on the amount of protection they provide (Table 1). We classified our annual growth measurements into six categories based on the cover class for the saguaro and the year in which the saguaro height was measured. We specified two time periods: between 1979 and 1994, a relatively wet period, and between 1995 and 2009, a dry period. Drezner [20,21] reports different growth rates in different locations with precipitation being an important factor. Using analysis of covariance with the R statistical package [22], we determined that there is an interaction between the cover class and time period that influenced growth based on height. Therefore, we used six models of growth from height based on the two time periods and three cover classes.

**Table 1:** Species of cover plants for saguaros in the census plots grouped into three classes.

Cover class	Scientific name	Common name	Saguaro count
**C1**	*Parkinsonia microphylla*	Foothill paloverde	403
	*Prosopis velutina*	Mesquite	99
	*Condalia warnockii*	Mexican crucillo	7
**C2**	*Acacia (Vachellia) constricta*	Whitethorn acacia	62
	*Larrea tridentata*	Creosote bush	46
	*Acacia (Senegalia) greggii*	Catclaw acacia	19
	*Ephedra* sp.	Mormon tea	11
	Other species		13
**C3**	*Isocoma tenuisecta*	Burroweed	79
	*Zinnia acerosa*	Desert zinnia	32
	*Psilostrophe cooperi*	Paperflower	12
	Grasses		17
	Other species		33
	open		31

*Note*: Cover classes: (C1) large trees; (C2) small trees or large shrubs; (C3) small shrubs, grass or plant with minimal or no cover. Saguaro counts include all new plants found under each species over the census period, 1942–2010.

**Fig 2:**
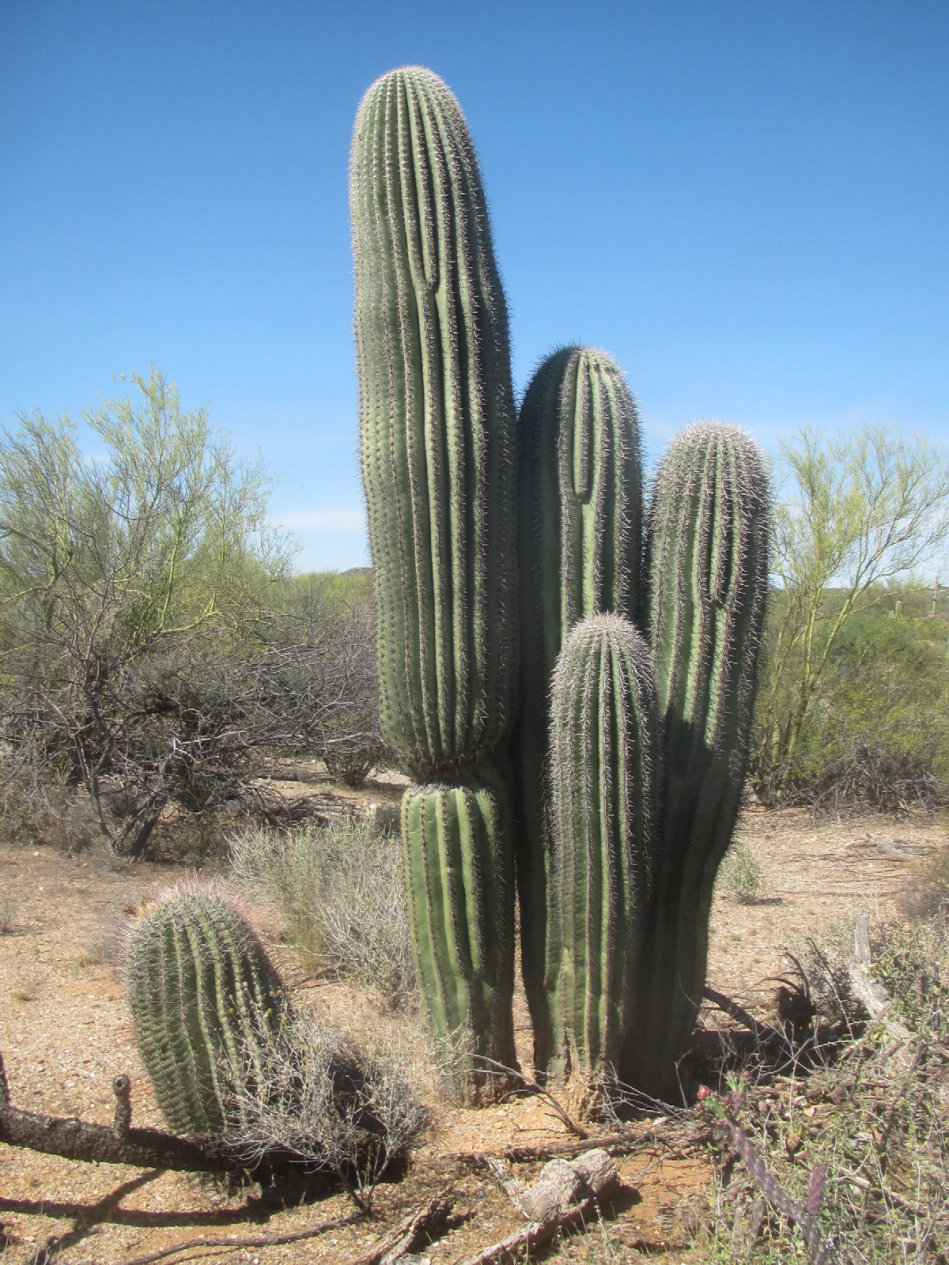
Photo taken in 2016 of four saguaros showing different growth rates. The saguaros were discovered in 1986 when they were 1 to 2.5 cm tall. The tallest saguaro in the photo is 287 cm tall and was 1 cm tall in 1986.

To estimate age from height requires a three-step process. First, develop a model of growth from height (we use log(growth) from log(height)) (S1 File, S1 Table). Then, use the model to develop a table to estimate height for a given age (S2 Table), and, finally, for each new saguaro, use the table to estimate its age based on its height. Subtracting the age estimate at the time of its discovery from the year of discovery gives an estimated germination year for each of the 874 newly found plants. We used Steenbergh and Lowe’s Table 2–10 [8] to estimate age from height of plants less than 4 cm tall when found. We use the height age tables (S2 Table) only to estimate age when a small saguaro is first discovered and not beyond that, because there is considerable variability in the growth rate of saguaros even when they are under the same nurse plant. For example, in 1986, four saguaros were discovered under a paloverde tree when they were between 1 cm and 2.5 cm tall and estimated to be 4 to 7 years old. By 2016, the four plants were 133 cm, 193 cm, 216 cm, and 287 cm tall (Fig 2). The smallest plant in 1986 (1 cm tall) grew to be the tallest plant in 2016 (287 cm tall).

**Fig 3:**
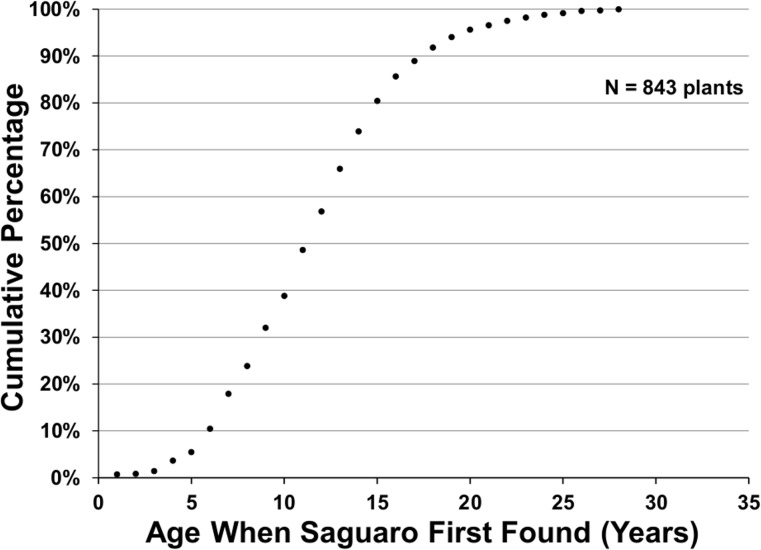
Cumulative percentage of saguaros discovered at ages four to thirty years. The age is based on the height of the saguaro in the year it is found.

Because of this variability in growth, the sooner a saguaro is discovered, the better the age estimate. In a natural setting, saguaros can take several years to reach the height of 2 cm (Table 2–2 in [8]), so there is a lag of several years between germination and when a saguaro can be discovered and its age estimated (Fig 3). Discovery was particularly challenging during the episode of regeneration, especially in the 1980s, because of the dense growth of annual plants and perennial grasses under nurse trees during that period. This dense growth under the nurse plants could be a contributing factor in the success of the saguaro establishment by providing additional shade, frost protection, and protection from discovery by herbivores [15].

### Saguaro reproductive potential

Pierson, Turner, and Betancourt [6] describe an index for saguaro reproductive potential based on the density of reproductive stem tips. Beginning in 1968, the number of branches, of at least 10 cm diameter, on each saguaro in all six plots was counted periodically. The main stem of all saguaros in height classes III, IV, and V (greater than 3.6 m tall) has the potential to produce flowers, as do their branches. In addition, most plants in height class II (1.8 m to 3.6 m tall) have the potential to flower, since flowering begins at around 2 m [23]. The number of plants in each height class (II, III, IV, and V) plus the total number of branches on those plants, then, is a count of the number of reproductive stem tips in the plot. The number of reproductive stem tips in the plot divided by the area of the plot gives the density of reproductive stem tips, which is the index of reproductive potential used by Pierson, Turner, and Betancourt [6]. Because we did not have branch data prior to 1968, reproductive potential in 1942 and 1960 was estimated based the number of plants in each height class (II, III, IV, and V) and an estimate of average number branches for plants in each height class using the 1968 branch data.

### Mortality

Mortality was analyzed in two ways: For the saguaros counted in the study plots in 1942, survival curves were calculated for cohorts defined by their height class in 1942. Survival curves for these plants show the percent of each 1942 cohort that survived each year from 1942 to the present. For saguaros found after 1942, we have a better estimate of age based on the height of the saguaro in the year it was found. So, we have good age estimates for each of these saguaros from the time of discovery until the time of death. Annual survival/mortality observations of 867 saguaros found in the plots after 1960 were used to construct survival curves for saguaros between the ages of 10 years and 40 years for each of three cover classes (Table 1). The data were analyzed using the Kaplan-Meier method [24] to compute the survival probabilities and the log rank test [25] to test the null hypothesis that there are no differences among the three cover classes in the survival curves.

## Results

### Height/age structure

**Fig 4:**
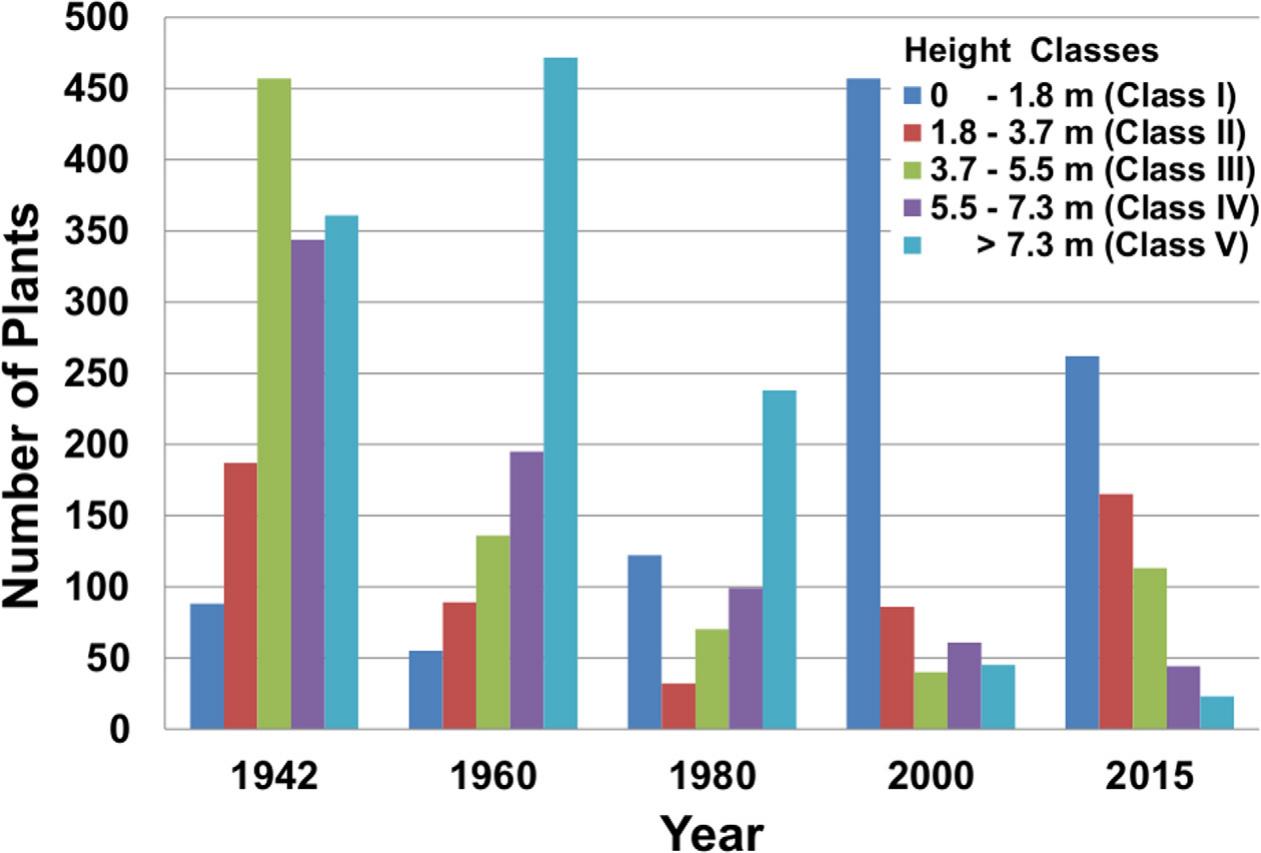
Height structure of the saguaro population in the census plots in the flats of the Saguaro National Park, Rincon Mountain District from 1942 to 2015.

The structure of the saguaro population in this study has been shaped by alternating periods of regeneration success or failure combined with ongoing mortality of older plants. The impact of this dynamic on the height/age structure of the population over the decades has been dramatic (Fig 4). The population in the six plots in 1942 was 1,437 plants (60 plants/hectare). The population in 2016 is 611 plants (25 plants/hectare). Initially, in 1942, the largest number of plants was in height class III (Fig 4), the height/age class where branching begins and flowering intensifies. The height/age structure in 1960 shows an aging population dominated by tall plants (50% of plants in height class V) with very few small (young) plants (in height class I). The height/age structure in 1980 is bimodal featuring relatively large numbers of small and large plants (height classes I and V). The height/age structure in 2000 shows a young population dominated by over 450 plants in height class 1 (< 1.8 m tall). The 2015 height/age structure is the mirror image of the 1960 height structure (Fig 4) except with fewer plants in each class. The population density in 2016 is less than half of the density in 1942. Only 34 plants of the 1,437 plants in the 1942 population are alive in 2016 (2.4%).

### An episode of regeneration: beginning, middle, and end

**Fig 5:**
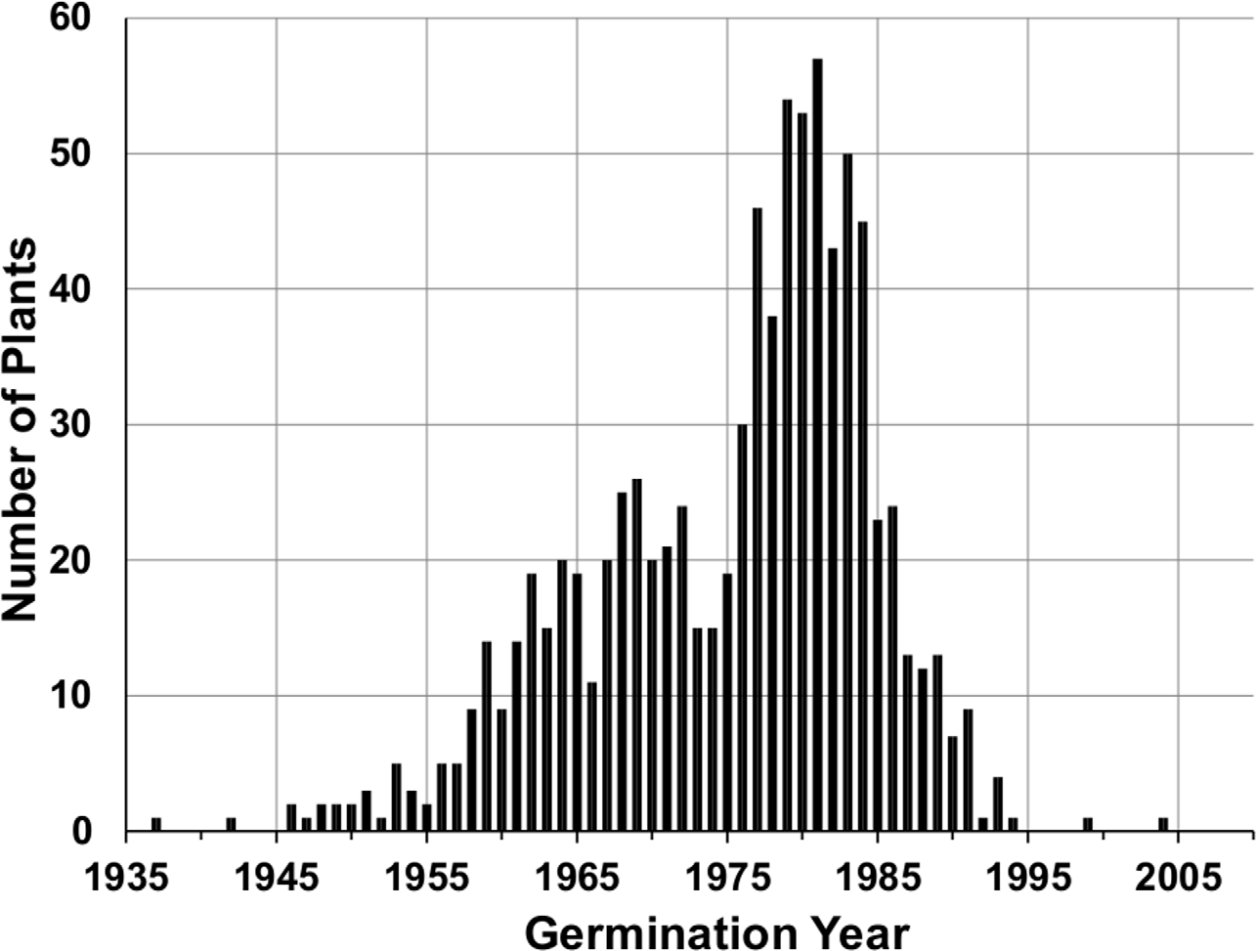
Saguaro population regeneration. The number of plants established each year based on plants found in the census plots during 1942–2015. The germination year was estimated based on the height in the year of discovery.

The number of plants established in each year from 1937 to 2002 was examined using all plants found in the study area after 1942 (Fig 5). Few of the discovered saguaros germinated prior to 1959. From 1959 through 1989, except for 1960, ten or more saguaros that grew to be discovered became established every year. We interpret 1959 as the beginning of a three-decade episode of population regeneration. The number of discovered plants establishing each year trended upward from 1959 to 1969 when it reached 26 plants per year (Fig 5). The middle of the episode of regeneration features a dip in the number of plants establishing in the years 1970 through 1975 where the establishment rate was below 20 plants per year. Establishment exceeded 40 plants per year in seven of the eight years from 1977 through 1984 (Fig 5).

There was a significant reduction in the number of plants establishing after 1984 (Fig 5) dropping to 9 plants in 1991. Only three plants have been found with estimated germination dates after 1993. An 11-cm-tall saguaro with a germination year of 1994 was found in 2007; a 6-cm-tall saguaro with a germination year of 1999 was found in 2009; and a 7-cm-tall saguaro with a germination year of 2004 was found in 2015. Because of the lag between germination and discovery, the end of the episode of recruitment takes a while to discover. Any plant germinating in 1998 would be 18 years old in 2016. Since over 80% of all plants discovered in the plots are age 18 years or younger when found (Fig 3), it is reasonable to assume we have found most of the plants that established between 1994 and 1998 and only one plant has been found for those establishment years. Hence, the decline in establishment observed in the early 1990s is real and the three-decade episode of establishment ended about 1993. Determining the length of the gap between episodes of regeneration awaits the experience of future censuses and depends on conditions being right for the next episode of regeneration.

### Reproductive potential

**Fig 6:**
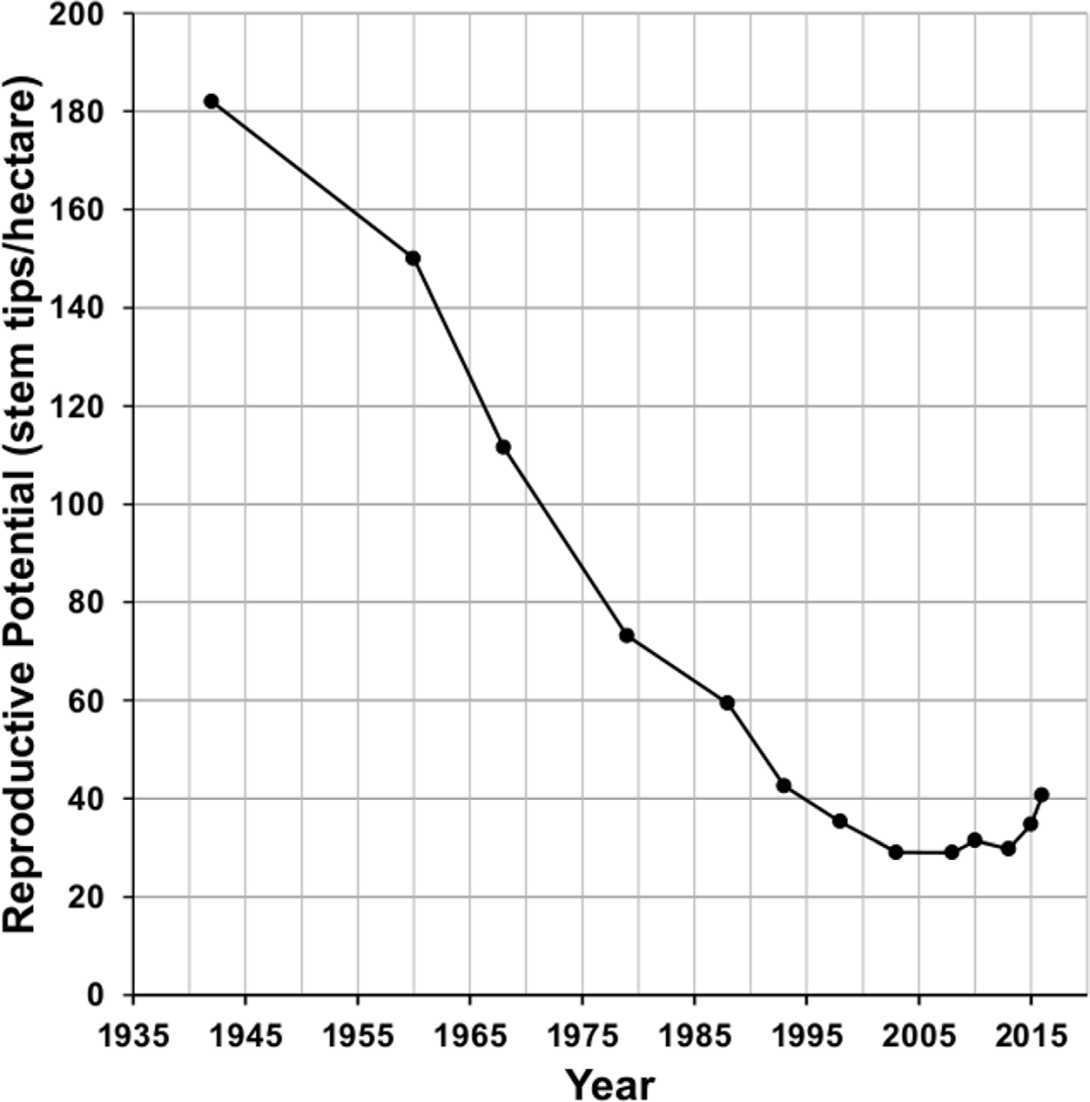
Saguaro reproductive potential from 1942 to 2016.

Reproductive potential, measured as the number of stem tips/hectare, depends on the height/age structure of the population, which has changed dramatically over the 75 years (Fig 6). The decline in the number of older, taller plants led to a decline in the reproductive potential in the plots for six decades from 1942 to 2003 (Fig 6). Reproductive potential declined from 80 to 63 stem tips/hectare during the period of peak regeneration (1977 to 1984). It declined sharply from 59 to 43 stem tips/hectare between 1988 and 1993 and leveled off in 2003 at about 30 stem tips/hectare. Reproductive capacity increased for the first time in 2010. Except for a dip in 2012 following the 2011 freeze mortality, reproductive potential has continued to increase through 2016 as plants from the episode of regeneration (1959 to 1993) reached reproductive size and began to grow branches. Reproductive potential in 2016 is 40 stem tips/hectare or about 50% of the 1977 reproductive potential and 63% of the 1984 reproductive potential which occurred during the period of peak regeneration.

### Mortality

**Fig 7:**
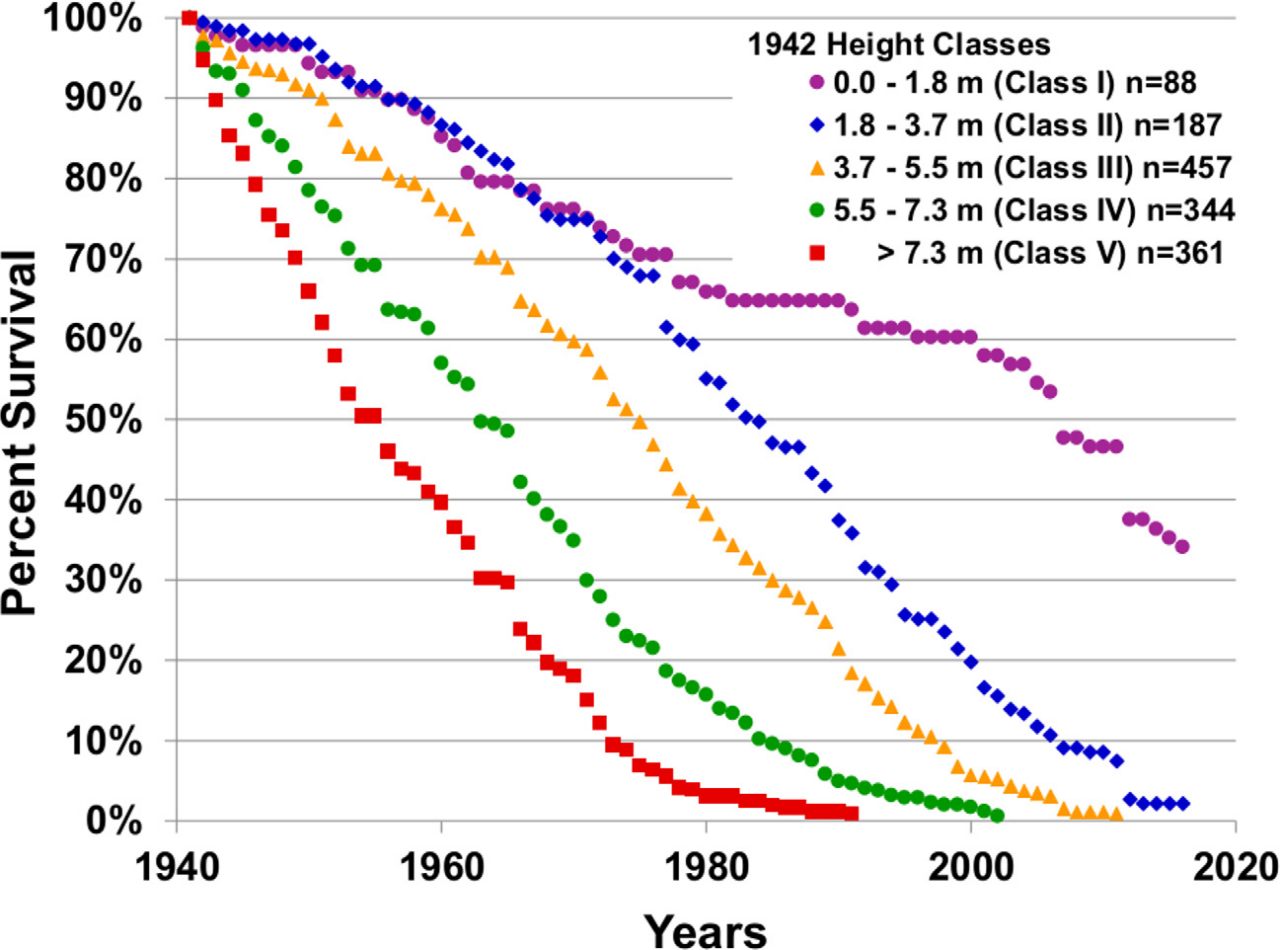
Survival curves from 1942 to 2016 for saguaros in height classes I through V in 1942.

The mortality of the 1942 population was strongly dependent on the height (age) of the plants in 1942 (Fig 7). For example, 50% of the plants in height class V (> 7.3 m) in 1942 (n = 361) were dead by 1954, whereas 50% of the plants in height class IV (5.5 m to 7.3 m) in 1942 (n = 344) were dead by 1963. Similarly, 50% of the plants in height class III (n = 457), height class II (n = 187) and height class I (n = 88) were dead by years 1975, 1988, and 2007 respectively. The median survival time is based on the year when percent survival is 50%. The median survival time for the 1942 height classes V, IV, III, II, and I was 12, 21, 33, 46, and 65 years after 1942 respectively. The last surviving plant in 1942 height classes V, IV, and III died in 1995, 2008, and 2011 respectively. Four plants from height class II in 1942 and 30 plants from height class I are still alive in 2016. So, in general, the taller/older plants in 1942 died sooner. Note that height class I is a broader age class than height classes II, III, and IV because the height classes are spaced equally at 1.8 m intervals, but the plants under 1.8 m tall grow more slowly than the taller plants. The five survivorship curves can be thought of as different expressions of a single survivorship curve by five age cohorts over seven decades. The figure shows the survivorship response of each cohort experiencing the varying climatic conditions of the decades (wet or dry, many catastrophic freezes or few etc.).

We do not have good age estimates for the height classes in 1942. For an approximation, we can use the median ages of plants in those height classes in 2015. We have fairly precise age estimates for plants discovered after 1958 based on their height at the time of discovery. The median age of plants in 2015 in height classes V, IV, III, II, and I is >85, 73, 51, 41, and 34 years respectively. Note that faster growing, and thus, younger plants will enter the taller height classes first and be followed by the older plants. Therefore, height class III, in particular, could contain a higher proportion of younger faster growing plants from the episode of regeneration (1959 to 1993) than height class III plants in, for example, a decade from now. So, the median age estimate for the height class III in 1942, based on the current height class III ages may somewhat low.

Adding the median survival time (Fig 7) to an estimate of the median age of plants in each cohort gives an estimate of the median age at death for the 1942 height classes as >98 years (V); 95 years (IV); 85 years (III); 83 years (II); and 99 years (I). An estimate of the age at death of the last plant to die in 1942 height classes V, IV, and III is >135, 134, and 121 years respectively.

Catastrophic freeze events are defined by Bowers as events where the temperature minimum is between -8.3 C and -5.6 C, the duration of the freeze is 15 to 20 hours, and there is widespread frost damage to Sonoran Desert plants [26]. There were four such freezes between 1946 and 1979. Between 1979 and 2011 there were no catastrophic freeze events of the type described by Bowers. On February 2^nd^ and 3^rd^, 2011 there was a freeze in the Tucson area that met the Bower’s criteria. In the 2012 census, following the 2011 freeze, there was a sharp drop in the percent survival of the 1942 height class I plants (Fig 7). The difference in mortality among age groups following the 2011 freeze is striking (Table 2). All four plants that were in 1942 height class III died and 9 of 14 (64%) of the 1942 height class II plants died in the year following the 2011 freeze. In contrast, fewer than 2% of the plants that were new to the plots after 1946 and were alive in 2011 died in the year following the freeze. None of the 270 plants between the ages of 29 and 80 years in 2011 died in the year following the freeze.

**Table 2:** Mortality following the freeze of 2011 as recorded in the Spring 2012 census.

Height/Age Class	Age in 2011 (Years)	Dead (#)	Survived (#)	Mortality
**Height class III in 1942**^**1**^	107 to 138	4	0	100%
**Height class II in 1942**^**2**^	100 to 126	9	5	64%
**Height class I in 1942**^**3**^	80 to 120	8	33	20%
**New in plots 1946–1961**	60 to 80	0	15	0%
**New in plots 1962–1990**	29 to 69	0	255	0%
**New in plots 1991–2011**	12 to 44	8^4^	314	2%
**Total**		29	623	4%

^1^3.6 to 5.4 m tall in 1942

^2^1.8 to 3.6 m tall in 1942

^3^ 0 to 1.8 m tall in 1942

^4^The eight dead plants that were new in the plots 1990–2011 were 20 to 30 years old. Five of the eight had poor cover (cover class C3) and all eight were growing slowly in the years prior to the freeze.

The age range of plants in the plots prior to 1962 in Table 2 required different estimation methods than the plants found after 1962. The low end of the age range for plants found between 1946 and 1961 was estimated at 60 years old because that group of plants included two plants that were 2.5 cm tall in 1958 when found. We estimate their year of germination to be 1951 and hence they were about 60 years old in 2011. The plants found in 1946 were not measured in the year they were found, but being found in 1946 means they were overlooked prior to 1946. Since we found 80% of our new plants by age 15 years old, we estimate these plants were no older than 15 years at the time they were found in 1946. That would put an early date of germination for them at 1931 and hence a maximum age of 80 in 2011. The age ranges for the 1942 height classes shown in Table 2 are based on a typical age range for plants in those height classes, which we have estimated based on the known ages of plants in those height classes in 2015.

Saguaro survival during a catastrophic freeze also depends on cover as well as age. Twenty-one species of plants serve as cover plants in our study plots. We have grouped them into three classes: C1) large trees (paloverde and mesquite), C2) small trees and large shrubs, and C3) small shrubs, grasses, and plants that provide minimal cover, or no cover plant at all (Table 1). Five of the eight small saguaros that died after the 2011 freeze were in cover class C3. There is indirect evidence that catastrophic freezes influenced the cover class composition of saguaros that survived long enough to be found among those that germinated prior to December 1978. There were three catastrophic freezes (January 1962, January 1971, and December 1978) between 1959 and 1978 [26] in the first part of the episode of regeneration, compared with none between 1979 and 1993 in the second part of the episode of regeneration. Between 1959 and 1978, only 65 out of 415 saguaros (16%) established under cover class C3 (poor cover) compared with the time between 1979 and 1993, a period of no catastrophic freezes, when 140 out of 410 saguaros (34%) established under cover class C3 (Χ^2^ = 53.2, df = 2, *p* < 0.001) (Table 3).

**Table 3:** The number of saguaros established under small shrubs, in the grass, or in the open (cover class C3) increased during the time of no catastrophic freezes after December 1978.

Cover Class	Established Before December 1978	Established After December 1978
**C1: Paloverde and mesquite**	292	191
**C2: Small trees and large shrubs**	57	80
**C3: Small shrubs, grass, or in open**	65	140
**Total**	415	410

*Note*: Tabular values include only those saguaros that germinated during the regeneration episode (1959–1993). The observed values are different from the expected values based on the chi-square test (*p* < 0.001).

**Fig 8:**
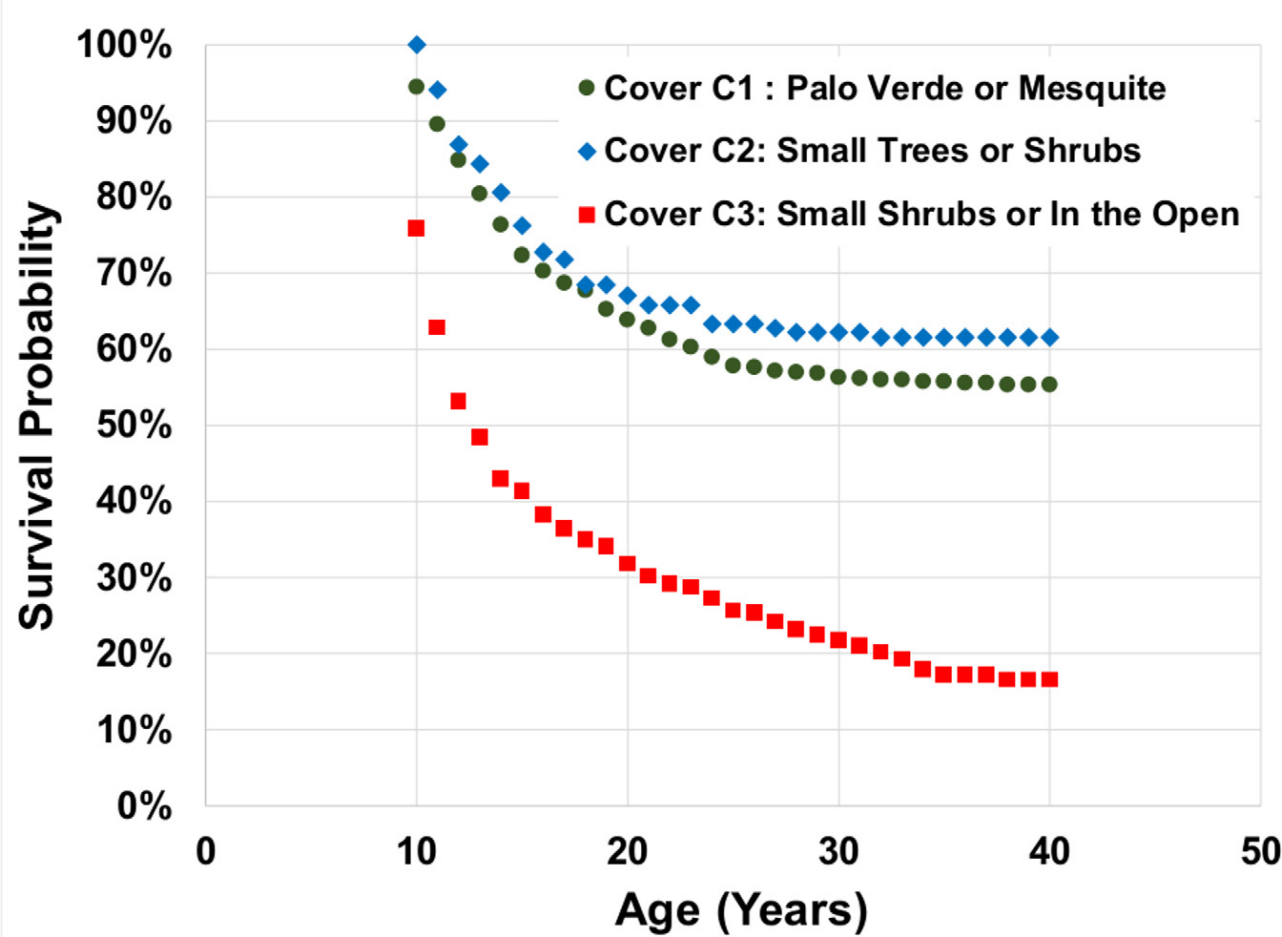
Kaplan-Meier survival probability curves for saguaros that were established during the episode of regeneration between 1959 and 1993.

Kaplan-Meier survival curves for plants 10 years and older that were established during the episode of regeneration also show that plant cover strongly influences survival (Fig 8). Survival of plants under paloverde or mesquite (cover class C1) and plants under small trees or large shrubs (cover class C2) after age 9 is significantly higher than survival under small shrubs or in the open (cover class C3) (log rank test Χ^2^ = 155, df = 2; *p* < 0.001). Overall, mortality of small saguaros in cover class C3 (62%) is significantly higher than mortality in cover class C1 (31%) and cover class C2 (21%) (Χ^2^ = 78.0, df = 2, *p* < 0.001) for saguaros that germinated during the episode of population regeneration (Table 4).

**Table 4:** Mortality through 2016 of saguaros that germinated during the episode of population regeneration that occurred from 1959 through 1993 based on cover class.

Cover Class	Dead (#)	Survived (#)	Mortality
**C1: Paloverde and mesquite**	148	335	31%
**C2: Small trees and large shrubs**	29	108	21%
**C3: Small shrubs, grass, or in open**	127	78	62%
**Total**	304	521	37%

## Discussion

The study reported here is unique in saguaro research. It is based on repeated annual observations of a large number of individual saguaros spanning three quarters of a century. The most striking features to emerge from the study are a 34-year episode of saguaro population regeneration and the relentless age-related mortality of the 1942 population. The episode of regeneration began about 1959 and gained momentum building to a peak between 1977 and 1984 (Fig 5). Population regeneration then tapered off to end the episode around 1993. During the period of regeneration, 828 new saguaros were added to the population. Since 1993, population regeneration has been very low (only three saguaros established). This pattern of mortality and regeneration has resulted in dramatic changes in the age structure of the population over time (Fig 4).

Alcorn and May in 1962 [27] predicted that, unless population regeneration occurred, the Cactus Forest in the Rincon Mountain District of Saguaro National Monument could lose all its saguaros by the year 2000. They were not far off. Only 34 of the 1437 plants counted in the six plots in 1942 are alive today (2%). To Alcorn’s great joy, he would learn a decade and a half after writing the 1962 paper that, as they were making their prediction in 1962, an episode of regeneration was beginning.

In 1963, Niering, Whittaker and Lowe [28] were pessimistic about the possibility of population regeneration in the Cactus Forest because of ongoing grazing there. They pointed to man-made causes–grazing and predator (coyote) control–rather than environmental events for the failure of new saguaros to become established. They saw cattle grazing decreasing the grasses and herbaceous ground cover, predator control increasing the rodent population, and rodents eating the very small saguaros before they could become established. They suggested that the damage in parts of the monument was largely irreversible. In part through their efforts, grazing was eventually curtailed in the Monument. The episode of regeneration reported here is evidence the impacts of grazing were not irreversible. During the peak of the period of regeneration, there was abundant perennial grass and winter herbaceous ground cover. On the other hand, since 1993, we have entered another period when new young saguaros are not being found. There is no grazing and no predator control, but an extended drought beginning in the late 1990s and extending to the present has greatly reduced the ground cover under the most common nurse plants (paloverde and mesquite).

### Population regeneration

Local and microhabitat circumstances influence saguaro population regeneration under the umbrella of region-wide climate patterns such as the El Niño Southern Oscillation (ENSO) [5,7], the Pacific Decadal Oscillation (PDO), and the North American Monsoon [29]. Pierson, Turner, and Betancourt [6] examined patterns of regeneration in saguaro populations at ten sites across the northern part of the Sonoran Desert to see, among other things, if the episodes of regeneration were synchronous and if they coincided with regional climate patterns. They found that the patterns of regeneration were complex, but generally that populations in the drier western part of the saguaro’s range tended to be less episodic than populations in the east [6]. Several plots in the Tucson area, including one near our study area, showed marked episodic recruitment, in contrast with a population on the south-facing rocky slopes of Sabino Canyon, near Tucson, that had a relatively stable population structure without strong episodic recruitment [6]. This is consistent with the observations of Steenbergh and Lowe [8] that populations in the rocky habitats of both the eastern and western divisions of the Saguaro National Park have a more stable height/age structure than populations in the flats. Collectively, these observations point to the fundamental importance of local conditions for saguaro recruitment.

Betancourt et al. [30] state that decades of frequent El Niño events occurred between 1900 and 1930 and again between 1960 and at least 1993. The period of frequent El Niño events after 1960 matches the time period for the episode of population regeneration reported here. This period also overlaps with an era from 1976 to 1993 when the PDO was positive, which favors wetter conditions in our area [29]. Swetnam and Betancourt [11] note that the period after 1976 was characterized by unprecedented annual growth rings in trees located in New Mexico. That period includes the time of maximum population regeneration in our plots (1977–1984). The episode of regeneration described here is not characterized by a single event or one good year, but rather a succession of favorable years that may depend on global climatic patterns. On the other hand, there was not an episode of population regeneration during the 1900–1930 period of frequent El Niño conditions, as noted below.

The contrast between our population and the population at Tumamoc Hill further illustrates the point that multiple local factors beyond broad regional patterns influence saguaro population regeneration. Pierson and Turner [7] reported on the saguaro population regeneration at the Desert Laboratory on Tumamoc Hill in Tucson, Arizona about 26 km west of the plots reported on here (Fig 1) based on censuses done in 1908, 1964, 1970, and 1993 of four ten-hectare plots. At Tumamoc Hill the largest increase in saguaro regeneration occurred during a wet period from 1930 through 1942 [7]. In contrast, there was almost no regeneration of the saguaro population in our study plots between 1930 and 1942, as indicated by the small number individuals in height class I (< 1.8 m) in 1942 and 1960 (Fig 4). On the other hand, Pierson and Turner [7] noted poor regeneration on all slopes during the late 19^th^ century, whereas in our plots, the large number of saguaros in height class III in 1942 suggests the late 19^th^ century was a particularly good period of regeneration based on the following method. Our estimate of the age range for height class III plants is younger and longer than an estimate based on deriving the age from the limits of the height class (3.6 to 5.4 m) alone. We base our age estimates on the height when the plant is found and follow its growth until it reaches that height class. Rapidly growing individuals reach height class III by age 38 years and the oldest member of 2015 height class III is 69 years old. Therefore, our estimate of the age of plants in height class III in 2015 range between 38 and 69 years old or roughly 40 to 70 years old. Using the Steenbergh and Lowe estimates for saguaros 3.6 m and 5.4 m tall (the height class limits) gives a narrower age range of 53 to 68 years [8]. If the large number of saguaros in the 1942 height class III plants had an age range similar to the 2015 height class III plants, they likely became established between 1870 and 1900 when Tumamoc Hill regeneration was low. We note that the numerous height class IV and V saguaros in 1942 would have established themselves earlier than 1870. Pierson, Turner, and Betancourt [6] state that across the northern Sonoran Desert region, regeneration rates were highest between 1780 and 1860, which is when many of the 1942 height class IV and V saguaros would have been established.

One of the ten plots studied by Pierson, Turner, and Betancourt [6] is in the flats of the Rincon Mountain District of Saguaro National Park near ours. Based on survivorship curves and height measurements taken in 1960, Pierson, Turner, and Betancourt [6] estimate a period between 1870 and 1970 where population regeneration was poor (less than what was needed to sustain the population at 1960 levels). Our estimate for our population using different assumptions and methodology is that there was a somewhat shorter period of very poor regeneration, roughly between 1900 and 1960, but still a very long period.

Pierson, Turner, and Betancourt [6] suggest that once a long period of poor regeneration has occurred, the pattern may be perpetuated, because it will be followed, after a lag, by a period of reduced reproductive potential. Reproductive potential was between 63 and 80 stem tips/hectare during the peak of the regeneration episode (Fig 6). Reproductive potential reached a minimum in our plots of 29 stem tips/hectare in 2003 and then rose for the first time in 68 years in 2010 and, except for 2012, has continued to rise. We expect it to continue to increase as the plants that were established at the peak of the episode of regeneration reach reproductive age and produce branches. The period of low reproductive potential in the plots has coincided with a period of extreme drought, so it is not possible to separate the effect of reproductive potential on regeneration independent of drought. However, the reproductive potential at its lowest is still only about 50% below the reproductive potential at the end of the peak of the episode of establishment. Because saguaro regeneration in the plots is spatially patchy, there are parts of the plots where the reproductive potential approaches the 60 reproductive stems/hectare level of the 1980s (51 stem tips/hectare in plot F-5 in 1998, for example) and yet there has been no regeneration in those areas since 1993.

We have described in the methods section the history of the plots. There are not sufficient controls or replications to assess the impact of the differences in plot history on regeneration or mortality, so we have not analyzed the plots separately. No obvious effects stand out. Subjectively, the impact of microhabitat factors such as soil type, topography (washes, ridges, slope, and aspect), and cover plant composition (e.g. presence or absence of paloverdes) are the primary influences on the patterns of regeneration and survival that we have observed, both within and between plots. The expansion of the road and picnic area into relatively good quality microhabitat could have influenced regeneration in one plot marginally.

Weiss, Castro, and Overpeck [31] contrast the drought of the 2000s with the drought of the 1950s and point out that temperatures during the drought of the 2000s have been generally higher than during the 1950s due to climate change. They note that the higher temperatures increase the evapotranspiration especially in the foresummer prior to the monsoons. Hence, we suspect drought, not reproductive potential, is primarily responsible for the lack of regeneration in this population in the current era. We observe a lack of establishment beginning about 1994. The drought of the 2000s actually began in 1996 [32]. We suspect that almost all saguaros germinating after 1993, but before the drought set in, died when they were 1 to 4 years old due to drought and associated herbivory before we could discover them. There was an isolated El Niño event in the 1997–98 water year. Our observations suggest that a single El Niño event embedded in a long-term drought is not sufficient to support saguaro regeneration at our site. Based on their 85-year study of saguaros at Tumamoc Hill near Tucson, AZ, Pierson and Turner [7] caution that wet periods do not always lead to saguaro regeneration due, at least in part, to high mortality among seedlings susceptible to freezing and drought.

### Mortality

It is widely accepted that a major cause of saguaro mortality is catastrophic freeze [8] and that mortality following a freeze can take more than one year to express itself. Bowers [26] analyzed nine catastrophic freezes that occurred in the Sonoran Desert between 1894 and 1979 and described three catastrophic freezes during the episode of population regeneration reported on here (January 1962, January 1971, and December 1978). Significant age-related mortality followed the freeze of February, 2011, but the most striking feature of the annual observations of mortality of the five height classes in 1942 is the steady year-to-year mortality and the strong age dependence in the survivorship curves (Fig 7).

There were no catastrophic freezes from 1979 through 2011, over 30 years, and yet age-related mortality continued apace (Fig 7). One way to pose the question is: What are the anatomical and physiological changes that occur in a saguaro as it ages that make it more susceptible to mortality? The columnar cacti are semi-tropical plants and, as the northern most columnar species, the saguaro is the most cold tolerant of the columnar cacti. Lowe and Steenbergh [33] suggest that cold events are exerting a selection pressure for cold tolerance on saguaro populations in the northern part of its range, which is where our plots are located. Any plant that lives for about a century is subjected to any number of stresses from the burrowing of the cactobrosis moth larvae [34], to root fungi [35], to the Saguaro Cactus Virus [36], to loss of spines, to bird holes, to the effects of drought, heat, and wind, and including non-catastrophic cold. Regardless of underlying cause, mortality in saguaros is most often expressed as bacterial necrosis, which is characterized by a wet black ooze from the cortical tissue and a distinctive smell. The pectolytic bacterium associated with bacterial necrosis is *Erwinia cacticida* [37], a bacterium with low contagion found associated with soft-rot on many species of cacti.

Johnson and Coppola [38] report that a major freeze occurred on January 14 and 15, 2007 in Tucson. Though not a catastrophic freeze in the sense of Bowers, it was the most significant cold event after the December 1978 catastrophic freeze. Although there was significant mortality recorded among the 1942 height class I plants in the spring census of 2007, this mortality occurred during the summer of 2006 and was not related to the 2007 freeze. The first catastrophic freeze in over thirty years was in February of 2011 [39].

The 2011 freeze event was characterized by an extended period of sub-freezing temperatures (about 15 hours the nights of February 2^nd^ and 3^rd^ with a high temperature of only 3.3 C on February 3^rd^), which is typical of a catastrophic freeze event [26]. Dimmitt [40], reporting a freeze in March 2013, suggested the freeze of 2011 was more damaging than the freezes of 2007 and 2013 because of wind that accompanied the 2011 freeze. The mortality data following the freeze of 2011 (Table 2) are noteworthy both from the saguaros that died (old plants and a few unthrifty small plants) and those that did not die. The high survival rate of saguaros under the age of 80 years suggests that saguaros have considerable freeze tolerance or are protected by microsite location and/or nurse plants [41]. Thus, a major portion of a saguaro population can survive a significant freeze event.

The impact of a catastrophic freeze on a saguaro population that has episodes of population regeneration will depend on the age structure of the population at the time of the freeze. Catastrophic freeze especially affects very young and very old saguaros. Nobel [41] reports that typically, very small saguaros are particularly vulnerable to freeze-related mortality mitigating microsite and nurse plant associations notwithstanding. The number of established plants germinating each year in our plots trends upward from 1960 through 1981, but there is an apparent dip in the trend from 1973 through 1975. This could well reflect the impact of the catastrophic freeze of December, 1978 on saguaros 3 to 5 years old at that time which led to an underestimate of establishment during 1973–1975. Each major freeze event will have its own signature, but the clear age-related mortality following the freeze event of 2011 (Table 2) suggests that the plants older than 80 years old were most susceptible to this freeze event. Not included in Table 2 is the observation immediately following the freeze of 2011 that a number of the branches that were 10 to 15 cm long on the height class III plants had turned black. We thought they might have been killed by the freeze. The following year, however, we observed almost none of the small branches had died. Instead, there was new growth extending beyond the dark areas and five years following the freeze it is now difficult to tell which branches were affected.

Survival curves for young plants (Fig 8) show that the type of plant cover makes a large difference in the probability of survival for a young saguaro. In addition to the survival curves showing the importance of plant cover, we observed that the number of saguaros established under less protective cover plants is dramatically different before and after the freeze of December, 1978 (Table 3). Most (70%) of the plants found that established prior to 1978 had cover class C1 (paloverde or mesquite). Prior to 1978, it is likely that there were factors that either were less conducive to establishment for saguaros under cover classes C2 or C3 or they did establish, but were killed by the freezes of 1962, 1971, and 1978 before they were found. The role of nurse plants and particularly shade in the establishment of young saguaros has been widely reported [15,41,42] and is confirmed by the survival curves of our young plants (Fig 8) and their faster growth (S2 Table) under paloverde and mesquite. Turner et al. [15] suggested that moderating high soil temperatures in the hot pre-monsoon summers was very important to the survival of young saguaros. Drezner [43] documented that paloverde offer protection from cold temperatures under their canopies. The lack of a severe freeze between 1978 and 2007 may have allowed the establishment of a large number of saguaros with cover classes C2 and C3. The 1980s also included years of abundant rain. The role of cover plants in maintaining soil moisture and lower soil temperatures may have been less critical during that time and more saguaros were able to establish under less cover. The dynamics of survival in relation to cover are different on the rocky slopes than in the flats where the study plots lie. Protective rocks and the aspect of slopes can have a positive impact on saguaro survival on rocky slopes [7,8,15].

With the single exception of the saguaro population at Tumamoc Hill [1,7], the saguaro population in Section 17 of the Cactus Forest in the flats of the Rincon Mountain District of Saguaro National Park is the longest and most studied saguaro population in the Sonoran Desert [44]. In 2011 and 2012, Don E. Swann led a huge volunteer effort to re-census, measure, and map using GPS all the saguaros in Section 17 –seventy years after the initial census in 1941–1942 [45]. They used the same 64 four-hectare plots that were established in 1941. Their effort provides a spatial context for the plots in our study, while our study provides a temporal context for theirs. The height/age structure for the entire section matches closely the age structure we observe in our six plots as shown for 2015 (Fig 4). The total population decline between 1942 and 2012 was 31% for the entire section compared with a decline of 56% for our six plots. Our plots were located within the Section in areas of larger decline. Their map of percent population change in the Section shows that 12 of the 64 four-hectare plots had a population increase over the 70 years [45]. Using 2012 height data of small plants and Steenbergh and Lowe’s height to age curves, Conver et al. [45] document the same episode of regeneration that we report here with peak establishment between 1978 and 1988.

### The long view

The saguaro plants that germinated in the 19^th^ century gave rise to a population that became established in the last half of the 20^th^ century. That population will be flowering and setting seed during much of the 21^st^ century. The observations made during the past 75 years in this study area suggest that the success of this population’s regeneration in the 21^st^ century will depend on a combination of factors including nurse plant quality, climate, and fire associated with the invasive non-native buffelgrass [6]. The optimal climatic conditions for saguaro population regeneration include a succession of good periods of rainfall (winter and summer) that build ground cover as well as good nurse plant protection for seedling germination and survival. Just as it was impossible to know in 1960 that three decades of regeneration lay ahead for this population of saguaros, it is not possible now to know what the next century will bring. We are not aware of another project based on an annual census of a perennial plant population spanning more than 75 years. Ongoing long-term studies, such as the one reported on here, are important to our understanding of environmental impacts on the Sonoran Desert in an era of climate change.

## Supporting Information

S1 File Age-height estimation procedures. The detailed three-step modeling protocol to estimate age from height is summarized.
(DOCX)

S1 Table Coefficients for the log growth–log height polynomial models. The coefficients were obtained by stepwise linear regression to model log growth (y) from log height (x) for saguaros between the ages of 8 years and 30 years.
(DOCX)

S2 Table Age-height models based on annual saguaro growth data during two time periods under three cover classes. These models are used to determine the age of saguaros based on their height at the time of discovery.
(DOCX)
