# Supplementary material for: Saguaro (Carnegiea gigantea) Mortality and Population Regeneration in the Cactus Forest of Saguaro National Park: Seventy-Five Years and Counting
Source: PLoS One. 2016 Aug 9;11(8):e0160899. doi: 10.1371/journal.pone.0160899 (PMC4978412; doi:10.1371/journal.pone.0160899)
Supplement: S1 File — The detailed three-step modeling protocol to estimate age from height is summarized. (DOCX) [file pone.0160899.s001.docx]

**S1 File. Age-height estimation procedures.**

The estimation of age from height utilized a three step process. First, a model of growth from height (we use log(growth) from log(height)) was developed. Then the model (S1 Table) was used to develop a table to estimate height for a given age, and, finally, for each new saguaro, we used the table (S2 Table) to estimate its age based on its height. Steenbergh and Lowe [8] used a seventh order polynomial equation to model log(growth) from log(height) and Drezner [20] used a tenth order polynomial equation. We followed Steenbergh and Lowe’s example and used stepwise linear regression of a seventh order polynomial using the R statistical package [22]. Specifically, we used the R linear model function “lm” with the step function “step” to determine the coefficients, a_i,_ in the following general equation where x is log (height) and y is log (growth):

$y=a_{0}+a_{1}x+a_{2}x^{2}+a_{3}x^{3}+a_{4}x^{4}+a_{5}x^{5}+a_{6}x^{6}+a_{7}x^{7}$ (1)

The coefficients (a_i_), shown in S1 Table, are used with Equation 1 for each cover-class time-period combination to determine the six log(growth) from log(height) models. The largest number of growth measurements came from saguaros in cover class C1 (saguaros growing under paloverde or mesquite) where we had 2,173 measurements in the first time period and 3,390 measurements in the second time period. There is a lot of variability in saguaro growth rates. The multiple R^2^ values were about 0.50 for most categories, but were lower for cover class C3, where growth rates are less predictable. Plots of residuals *vs* fitted values using the plot function in R [22] indicated the models were unbiased.

The log(growth)-log(height) models were then used to calculate the age-height values in an iterative manner (S2 Table): We start with the Steenbergh-Lowe [8] estimate of a height of 4 cm for an 8-year-old saguaro for all six models. Note that if this estimate of age 8 years for height of 4 cm is changed by a year or two, the effect is the same on all of the models and has the effect of shifting the germination year estimates of the entire population by a year or two. The annual growth estimate of a 4-cm tall saguaro is calculated using Equation 1 and that growth estimate is added to 4 cm to give the height at age 9 years. The procedure is repeated using the height of the 9-year-old plant to give the estimate of growth to be added to the height at age 9 years to give the height at age 10, and, so on, for the subsequent years of each model (S2 Table). We start at age 8 years for all six categories, because we have insufficient data on growth of saguaros smaller than 4-cm tall. Steenbergh-Lowe have the only known published data on age-heights of very small saguaros growing in a natural setting from germination to 8 years old [8]. The Steenbergh-Lowe data are from saguaros growing near the plots in the current study. The age-height estimates in S2 Table do not go beyond the age of 30 years because they are used only to estimate age based on height when a saguaro is found and almost all the saguaros found in our plots are found well before 30 years in age (95% are found by age 20 years). We prefer not to extend S2 Table to plants older than 30 years because such an extension is not needed for our purpose and as plants grow beyond thirty years, the impact of the cover classes is reduced because cover plants die (particularly the small cover plants and the palo verdes, but not the mesquites) and the impact of the time periods is reduced because older plants lives span the two time periods. The sole purpose of the Table S2 is to assign a germination year to each saguaro added to the plot.

**References**

All numbered references are available in the primary text.
