# Supplementary material for: Saguaro (Carnegiea gigantea) Mortality and Population Regeneration in the Cactus Forest of Saguaro National Park: Seventy-Five Years and Counting
Source: PLoS One. 2016 Aug 9;11(8):e0160899. doi: 10.1371/journal.pone.0160899 (PMC4978412; doi:10.1371/journal.pone.0160899)
Supplement: S1 Table — The coefficients were obtained by stepwise linear regression to model log growth (y) from log height (x) for saguaros between the ages of 8 years and 30 years. (DOCX) [file pone.0160899.s002.docx]

**S1 Table. Coefficients for the log growth – log height polynomial models**. The coefficients were obtained by stepwise linear regression to model log growth (y) from log height (x) for saguaros between the ages of 8 years and 30 years.

| **Model Coefficients** | **Time Period** | | | | | |
| --- | --- | --- | --- | --- | --- | --- |
|  | **1979 to 1994 (Wet Years)** | | | **1995 to 2009 (Dry Years)** | | |
|  | **Cover Class^1^** | | | **Cover Class^1^** | | |
|  | **C1** | **C2** | **C3** | **C1** | **C2** | **C3** |
| **a_0_** | -0.6973 | 0.1768 | 71.9678 | -0.2214 | 0.0682 | 0.0577 |
| **a_1_** | 9.8048 | 0 | -491.648 | 0 | 0 | 0 |
| **a_2_** | -37.7911 | 0 | 1379.2545 | 0 | 0 | 0 |
| **a_3_** | 69.4303 | 0 | -2069.4233 | 1.9545 | 0.1577 | 0 |
| **a_4_** | -67.8115 | 0.1536 | 1801.1092 | -2.5692 | 0 | 0.1170 |
| **a_5_** | 36.3946 | -0.0516 | -911.6401 | 1.2808 | 0 | -0.0304 |
| **a_6_** | -10.1162 | 0 | 248.9582 | -0.2235 | -0.0052 | 0 |
| **a_7_** | 1.1375 | 0 | -28.3433 | 0 | 0 | 0 |
| **Multiple R^2^** | 0.48 | 0.51 | 0.21 | 0.54 | 0.62 | 0.44 |
| **# of plants** | 340 | 70 | 86 | 342 | 119 | 146 |
| **# of observations** | 2167 | 382 | 233 | 3382 | 1163 | 1270 |

**^1^** Cover classes are based on species shown in Table 1 and described as (C1) large trees; (C2) small trees or large shrubs; (C3) small shrubs, grasses or plants with minimal or no cover.
