# Supplementary material for: Saguaro (Carnegiea gigantea) Mortality and Population Regeneration in the Cactus Forest of Saguaro National Park: Seventy-Five Years and Counting
Source: PLoS One. 2016 Aug 9;11(8):e0160899. doi: 10.1371/journal.pone.0160899 (PMC4978412; doi:10.1371/journal.pone.0160899)
Supplement: S2 Table — These models are used to determine the age of saguaros based on their height at the time of discovery. (DOCX) [file pone.0160899.s003.docx]

**S2 Table. Age-height models based on annual saguaro growth data during two time periods under three cover classes.** These models are used to determine the age of saguaros based on their height at the time of discovery.

| **Age (Years)** | **Saguaro Height (cm)** | | | | | |
| --- | --- | --- | --- | --- | --- | --- |
|  | **Time Period** | | | | | |
|  | **1979 to 1994** | | | **1995 to 2009** | | |
|  | **Cover Class^1^** | | | **Cover Class^1^** | | |
|  | **C1** | **C2** | **C3** | **C1** | **C2** | **C3** |
| **8** | 4.0**^2^** | 4.0**^2^** | 4.0**^2^** | 4.0**^2^** | 4.0**^2^** | 4.0**^2^** |
| **9** | 5.5 | 5.3 | 4.7 | 4.9 | 5.2 | 5.2 |
| **10** | 7.1 | 6.9 | 5.7 | 5.9 | 6.6 | 6.4 |
| **11** | 9.0 | 8.6 | 7.0 | 7.1 | 8.0 | 7.6 |
| **12** | 11.1 | 10.4 | 8.5 | 8.4 | 9.5 | 8.9 |
| **13** | 13.5 | 12.4 | 10.2 | 9.9 | 11.1 | 10.3 |
| **14** | 16.1 | 14.4 | 12.0 | 11.6 | 12.9 | 11.7 |
| **15** | 18.9 | 16.6 | 13.8 | 13.4 | 14.8 | 13.1 |
| **16** | 21.8 | 19.0 | 15.7 | 15.4 | 16.8 | 14.7 |
| **17** | 25.0 | 21.6 | 17.8 | 17.6 | 19.0 | 16.3 |
| **18** | 28.4 | 24.4 | 20.0 | 20.0 | 21.4 | 18.0 |
| **19** | 32.0 | 27.4 | 22.3 | 22.5 | 23.9 | 19.8 |
| **20** | 35.9 | 30.7 | 24.7 | 25.3 | 26.7 | 21.7 |
| **21** | 40.1 | 34.3 | 27.2 | 28.4 | 29.8 | 23.7 |
| **22** | 44.7 | 38.2 | 29.7 | 31.6 | 33.1 | 25.8 |
| **23** | 49.8 | 42.4 | 32.2 | 35.2 | 36.7 | 28.0 |
| **24** | 55.2 | 47.1 | 34.6 | 39.0 | 40.6 | 30.4 |
| **25** | 61.3 | 52.1 | 37.1 | 43.2 | 44.9 | 33.0 |
| **26** | 67.9 | 57.7 | 39.4 | 47.7 | 49.6 | 35.7 |
| **27** | 75.2 | 63.7 | 41.8 | 52.7 | 54.8 | 38.6 |
| **28** | 83.2 | 70.3 | 44.2 | 58.1 | 60.4 | 41.7 |
| **29** | 91.8 | 77.5 | 46.6 | 64.0 | 66.6 | 45.0 |
| **30** | 101.8 | 85.3 | 49.1 | 70.6 | 73.4 | 48.6 |

**^1^** Cover classes are based on species shown in Table 1 and described as (1) large trees; (2) small trees or large shrubs; (3) small shrubs, grasses or plants providing minimal or no cover.

**^2^**All six models begin with an initial height of 4 cm at age 8 based on the analysis by Steenbergh and Lowe [8] of the growth of very small saguaros.

**References**

All numbered references are available in the primary text.
